# Aromatase inhibitors in men: effects and therapeutic options

**DOI:** 10.1186/1477-7827-9-93

**Published:** 2011-06-21

**Authors:** Willem de Ronde, Frank H de Jong

**Affiliations:** 1 Department of Internal Medicine, Kennemer Gasthuis, P.O. Box 417, 2000 AK Haarlem, The Netherlands; 2 Department of Internal Medicine, Erasmus university Medical Center, P.O. Box 2040, 3000 CA, Rotterdam, The Netherlands

## Abstract

Aromatase inhibitors effectively delay epiphysial maturation in boys and improve testosterone levels in adult men Therefore, aromatase inhibitors may be used to increase adult height in boys with gonadotropin-independent precocious puberty, idiopathic short stature and constitutional delay of puberty. Long-term efficacy and safety of the use of aromatase inhibitors has not yet been established in males, however, and their routine use is therefore not yet recommended.

## Background

Over the past 15 years it has become evident that in men estradiol is responsible for a number of effects originally attributed to testosterone. Estradiol has an important role in gaining and maintaining bone mass, closing of the epiphyses and the feedback on gonadotropin secretion. This fact became particularly evident in men with aromatase deficiency. Aromatase is the enzyme responsible for conversion of androgens to estrogens. Men with estrogen deficiency caused by a mutation in the CYP19 gene suffer from low bone mineral density (BMD) and unfused epiphyses, and have high gonadotropin and testosterone levels [1]. Estrogen excess in turn has been associated with premature closure of the epiphyses, gynecomastia and low gonadotropin and testosterone levels. Lowering estrogen levels in men has emerged, consequently, as a potential treatment for a number of disorders including pubertas praecox, the andropause (also referred to as late-onset hypogonadism) and gynecomastia. Aromatase inhibitors were proven to be safe, convenient and effective for the treatment of hormone sensitive breast cancer in women although their use is associated with a modest increase in bone resorption [2,3]. This review will discuss the potential targets and the evidence for the use of aromatase inhibitors in men and adds more recent data to the text of an earlier review on this subject [4].

### Metabolism of estrogens in men

Aromatase, also known as estrogen synthetase, is the key enzyme in estrogen biosynthesis. The enzyme, localized in the endoplasmic reticulum of the estrogen-producing cell, is encoded by the *CYP19A1 *gene. This gene is a member of the *CYP *gene family, encoding a class of enzymes active in the hydroxylation of endogenous and exogenous substances. The *CYP19A1 *gene is localized on chromosome 15 and comprises nine exons; the start codon for translation is located on exon 2. Transcription of the aromatase gene is regulated by several tissue-specific promoters. These promoters are under the influence of different hormones and growth factors such as gonadotropins (gonadal promoter II) and interleukin-6, interleukin-11 and tumor necrosis factor-a (adipose/bone promoter I.4; for review see [5]). Aromatase activity has not only been demonstrated in gonads and placenta but also in brain [6], fat tissue [7,8], muscle [9], hair [10], bone [11] and vascular tissue [12].

Estradiol is the most potent estrogen produced in the body. It is synthesized from testosterone or estrone via aromatase or 17β-hydroxysteroid dehydrogenase, respectively. The total estradiol production rate in the human male has been estimated to be 35-45 μg (0.130-0.165 μmol) per day, of which approximately 20% is directly produced by the testes [13,14]. Roughly 60% of circulating estradiol is derived from direct testicular secretion or from conversion of testicular androgens. The remaining fraction is derived from peripheral conversion of adrenal androgens [15]. The mean estradiol plasma concentration in men is only about 1/200 of the mean plasma testosterone concentration [16] and is comparable to estradiol levels found in women in the early follicular phase of the menstrual cycle.

### Phenotype of aromatase deficiency and excess

To date, eight males with aromatase deficiency have been described: seven adults [17-23] and one newborn [24]. Estradiol levels in these males were extremely low. All adult aromatase-deficient men demonstrated a remarkably low bone mass and unfused epiphyses leading to linear growth into adulthood and above-average body length. Testicular size in these men ranged from micro- to macroorchidism and the plasma testosterone levels varied roughly in accordance with testis size. Levels of luteinizing hormone (LH) were either normal or elevated. Four men were infertile, in one younger male fertility was not described. Two aromatase-deficient men had a brother who also suffered from infertility despite a normal aromatase genotype, suggesting an unrelated second condition. Once treated with estradiol, epiphyses closed, BMD increased and disturbances in the lipid profile improved in most of these patients.

On the other hand, several, mostly familial cases of aromatase excess have been reported. The clinical picture consists of gynecomastia, accelerated growth and premature bone maturation due to excessive peripheral estrogen synthesis. Stratakis et al. [25] described a family with aromatase excess syndrome in which the syndrome appeared to be caused by inappropriately high expression of an alternative first exon. Shozu et al. [26] described a father and his son and one unrelated patient with aromatase excess caused by a chromosomal rearrangement, which placed the aromatase gene adjacent to cryptic promoters. As a result an inappropriate amount of aromatase was expressed in adipose tissue of the affected subjects.

These case reports illustrate the important contribution of estrogens to male health and identify the possible indications and risks of aromatase-inhibitor treatment in men. Aromatase inhibitors may be used to treat or prevent gynecomastia. They may be used to increase gonadotropin secretion and thereby stimulate Leydig and Sertoli cell function. Aromatase inhibitors may be used to prevent or delay epiphysial closure and thereby increase adult height. A major concern of aromatase inhibition is the possible detrimental effect on bone mineralization.

### Aromatase inhibitors

Aromatase inhibitors are classified as either steroidal or nonsteroidal, or as first, second or third generation. Steroidal inhibitors such as formestane and exemestane inhibit aromatase activity by mimicking the substrate androstenedione. Nonsteroidal enzyme inhibitors such as anastrozole and letrozole inhibit enzyme activity by binding with the heme iron of the enzyme. First-generation aromatase inhibitors such as aminoglutethimide are relatively weak and nonspecific; they can also block other steroidogenic enzymes necessitating adrenal steroid supplementation. Third-generation inhibitors such as letrozole and anastrozole are potent and do not inhibit related enzymes. They are well tolerated and apart from their effects on estrogen metabolism their use does not appear to be associated with important side effects in postmenopausal women [27]. Although aromatase inhibition by anastrozole and letrozole is reported to be close to 100%, administration of these inhibitors to men will not suppress plasma estradiol levels completely. In men third-generation aromatase inhibitors will decrease the mean plasma estradiol/testosterone ratio by 77% [28,29]. This finding probably relates to the high plasma concentrations of testosterone, a major precursor for estradiol synthesis in adult men. As aromatase inhibition is dose dependent it has been suggested that aromatase is less suppressed in the testis compared to adipose and muscle tissue, explaining the incomplete efficacy of aromatase inhibition in men. Aromatase activity is high in the testes and the molar ratio of testosterone to letrozole is much higher in the testes compared with adipose and muscle tissue. When testicular testosterone and estradiol synthesis are suppressed and testosterone is administered exogenously in combination with letrozole, however, the estradiol/testosterone ratio is suppressed by 81% [30], which is only marginally different from the suppression of this ratio in intact men after treatment with letrozole. This incomplete suppression may be regarded as advantageous for it prevents excessive reduction of estrogen levels in men and the possible associated adverse effects. In postmenopausal women with breast carcinoma, long-term use of potent aromatase inhibitors reduces circulating estradiol levels by 88% [31] and is associated with adverse effects on bone [2,3]. Due to the much higher estrogen levels in treated men it remains to be determined whether this also holds true for men.

### Effects of aromatase inhibition on luteinizing hormone release and testosterone production

It is well known from experimental evidence and from clinical observations that estradiol has powerful effects on gonadotropin release in men. Modulation of plasma estradiol levels within the male physiological range is associated with strong effects on plasma levels of LH through an effect at the level of the pituitary gland [32]. Lowering estradiol levels, by administering an aromatase inhibitor, is associated with an increase in levels of LH, follicle-stimulating hormone (FSH) and testosterone [28,29]. Aromatase inhibitors, therefore, have been suggested as a tool to increase testosterone levels in men with low testosterone levels. Due to their mode of action the use of aromatase inhibitors is limited to men with at least some residual function of the hypothalamo-pituitary-gonadal axis. Therefore aromatase inhibitors have been tested in older men suffering from so-called late-onset hypogonadism or partial androgen deficiency. Aging in men is associated with a gradual decline of total and free testosterone levels [33] as a result of combined testicular and hypothalamic dysfunction. The decline of testosterone levels has been implicated in the pathogenesis of physical frailty in older men. Androgen treatment, therefore, has been advocated for older men with signs and symptoms of androgen deficiency and unequivocally low plasma testosterone levels [34,35].

Aromatase inhibitors may be an attractive alternative for traditional testosterone substitution in elderly men because these compounds can be administered orally once daily and may result in physiological 24 h testosterone profiles. Additionally, misuse of aromatase inhibitors is unlikely since testosterone levels will not be stimulated to vastly supraphysiological levels. A small, controlled study demonstrated that anastrozole in a dose of 1 mg daily during 12 weeks will result in doubling of the mean bioavailable testosterone level in older men [36]. A more recent study also showed a moderate but significant effect of aromatase inhibition on estradiol and testosterone levels in older men [37]. Treatment with atamestane 100 mg once daily resulted in a 40% increase in total testosterone levels after 36 weeks. However, no beneficial effects were seen on muscle strength, body composition or quality-of-life scores. A similar increase of testosterone levels in the absence of effects on body composition and strength was reported in a study, in which elderly men with borderline low levels of serum testosterone were treated with anastrozole during 1 year [38]. There is a number of possible explanations for the lack of a clear treatment effect. First of all, the numbers of studied subjects were relatively small. Moreover, the mean baseline testosterone levels in the treated groups were in, or only slightly below, the normal range for young adult men and the relative increase in testosterone levels may have been too small. It has been suggested that men with the lowest baseline testosterone levels benefit most from testosterone substitution [39]. Finally, the decreased levels of estradiol may have affected the expected rise in lean body mass [38]. These observations outline a serious limitation of the use of aromatase inhibitors in older men; the stimulating effect on testosterone levels may be too weak, especially in the men with the lowest baseline testosterone levels who would potentially benefit most.

### Effects of aromatase inhibition in obese men

Peripheral androgen aromatization is enhanced in subjects with increased body mass index [40]. Massively obese men show markedly increased plasma estradiol concentrations and low testosterone concentrations [41]. In three small studies, letrozole or testolactone has been administered to morbidly obese men to improve their testosterone levels [42-44]. Treatment resulted in normalization of testosterone levels in all subjects, with a concomitant suppression of the originally increased levels of estradiol. This normalization of the estradiol/testosterone ratio might be of advantage, because of the suppressive effects of testosterone on the expression of the estrogen receptor β, which in itself, in the presence of high levels of estradiol, can suppress the expression of GLUT-4, leading to insulin insensitivity [45]. A case study describes a morbidly obese infertile man, who after a similar treatment with anastrozole showed a normalized pituitary-testis axis, spermatogenesis and fertility [46]. However, testosterone levels will also improve on weight loss [47], which is the intervention of choice for obese men with or without low testosterone levels.

### Effects of aromatase inhibition on release of follicle-stimulating hormone and spermatogenesis

Although FSH release is primarily under the control of inhibin, circulating estradiol has a substantial effect on FSH levels in men [28]. Aromatase inhibition results in a three-fold increase in levels of FSH [28,29] in eugonadal men and may potentially stimulate sperm production. Earlier studies using tamoxifen or clomifene to increase FSH levels did not show unequivocal evidence for the efficacy of this approach [48]. Uncontrolled studies using anastrozole, testolactone or letrozole have shown some evidence for a positive effect on sperm concentration and motility [49-51]. However, one double-blind crossover trial using testolactone did not show a significant improvement of sperm quality in men with oligospermia [52]. More recently, a study in which anastrozole was added to the treatment with tamoxifen in men with idiopathic oligoasthenoteratozoospermia and a decreased testosterone over estradiol ratio after treatment with tamoxifen alone indicated an increased pregnancy rate compared with the group without the addition of the aromatase inhibitor [53]. Finally, pretreatment with aromatase inhibitors was described to lead to positive results of testicular sperm extraction in Klinefelter's syndrome patients with low pretreatment testosterone concentrations: men from whose testes spermatozoa were retrieved showed higher posttreatment testosterone levels and testosterone over estradiol ratios compared to men in whom no spermatozoa could be obtained, whereas pretreatment levels of testosterone, LH and FSH did not predict the result of treatment outcome [54].

### Effects of aromatase inhibition on bone metabolism and epiphysial closure

Estrogens are essential for epiphysial maturation in boys. Aromatase inhibitors, therefore, may be used to lower estradiol levels and thereby slow down epiphysial maturation. This approach proved successful in rare conditions such as the aromatase-excess syndrome [25] and high estrogen levels due to Sertoli cell tumors in boys with Peutz-Jeghers syndrome [55]. In boys with familial male precocious puberty due to activating mutations of the LH receptor, also known as testotoxicosis, treatment with an antiandrogen in combination with an aromatase inhibitor to prevent effects on bone is the treatment of choice. In an earlier study a combination of spironolactone and testolactone proved effective [56], whereas in later studies the combination of bicalutamide and anastrozole was used [57-59].

Aromatase inhibition has also been studied in boys with idiopathic short stature. Boys with a mean age of 11 years at the start of the study were treated with letrozole 2.5 mg once daily or placebo for 2 years [60]. Letrozole treatment was associated with higher plasma levels of gonadotropins and testosterone in boys who entered puberty during the study. In spite of this fact, plasma estradiol levels were mostly lower in the letrozole-treated group. Both groups showed similar growth velocity but bone age progressed significantly slower in the letrozole group resulting in a gain of 5.9 cm in predicted adult height. The fact that growth velocity was not affected is remarkable in the light of the observation, that in adult men treated with a combination of testosterone and anastrozole the responses to GH secretagogues were smaller than in men treated with a combination of testosterone and a placebo; the GH and IGF-1 concentrations were positively correlated with estradiol levels [61]. Also in letrozole-treated boys in whom treatment started at the beginning of puberty, IGF-I levels were lower than in placebo-treated controls [62]. As expected, GH-deficient boys treated with GH and anastrozole showed a larger increase in height than their GH only-treated controls [63].

Boys with constitutional delay of puberty are typically small for their age and final adult height is often in the low-normal range. These boys may be treated with androgens to induce puberty. Although testosterone induces growth velocity, the estrogens aromatized from testosterone will accelerate epiphysial maturation and for that reason reduce adult height further. The combination of testosterone and letrozole, therefore, was tested in boys with constitutional delay of puberty. This combination treatment effectively increased growth velocity but epiphysial maturation was slower in the letrozole-treated group, leading to a significant increase in predicted adult height [64,65].

### Effects of aromatase inhibition on male breast

Aromatase inhibitors are widely prescribed for hormone-responsive breast carcinoma in postmenopausal women. It is well known that aromatase inhibition results in a dramatic reduction of tumor estrogen concentrations [66]. As gynecomastia in men presumably results from an imbalance between androgen and estrogen action, aromatase inhibition was tested as a treatment for gynecomastia in boys. Treatment with anastrozole daily for 6 months, however, did not result in a significant improvement compared with placebo [67]. This is in accordance with the data summarized in a recent review [68], describing similar responses to placebo, tamoxifen and anastrozole in a number of observational studies. Anastrozole was also studied in a group of prostate cancer patients treated with bicalutamide, an androgen antagonist. A dose of 1 mg daily appeared to be mildly effective against the appearance of gynecomastia. Tamoxifen was much more effective, however, in the prevention of gynecomastia in these men [69,70]. Due to these disappointing results, aromatase inhibitors are not recommended as a first-line treatment for gynecomastia in men.

Data on treatment of male mammary tumors with aromatase inhibitors are scarce and indicate that this treatment modality is unlikely to be successful because of the unwanted effect of increased levels of testosterone, making it impossible to reach the low estradiol levels obtained in postmenopausal women after this treatment [71]. The combination with a GnRH analog in order to prevent this increase did not yield beneficial results either [72].

### Safety and concerns for aromatase inhibitors in men

Extensive experience with third-generation aromatase inhibitors in postmenopausal women did not reveal major side effects related to their use. Long-term use in postmenopausal women is associated with a moderate increase in bone resorption and a modest decrease in BMD compared with placebo [2,3]. As outlined above, low BMD is a characteristic sign of aromatase deficiency but also in normal men most cross-sectional studies showed that bioavailable or total estradiol levels are associated with BMD [73-77]. The primary concern, therefore, associated with aromatase inhibition in men is the negative effect it may have on bone metabolism. In most studies utilizing aromatase inhibitors in men estradiol levels decreased only moderately. Additionally, the suppression of plasma estradiol levels in men is associated with an increase in gonadotropin levels, which stimulate the production of testosterone, the main precursor for estradiol synthesis. Khosla et al. [76,78] proposed a threshold for bioavailable estradiol of 30 pM, below which BMD appeared to be strongly and negatively associated with the plasma bioavailable estradiol concentration in men. Thresholds should be interpreted with great caution because they rely heavily on the methods used to measure total or bioavailable estradiol levels. These authors used ammonium sulfate precipitation to measure bioavailable estradiol levels whereas if they had calculated bioavailable estradiol levels using the popular Sodergard equation [79,80] their proposed threshold may have been as high as 75 pM. In experimental settings, selective withdrawal of estradiol in men was associated with an increase in markers of bone resorption [30,81]. In the studies published so far aromatase inhibition in men did not appear to be associated with adverse effects on bone in a number of studies [37,59,82,83], but in a more recent study a decrease of spine BMD was observed after one year of treatment of elderly men with anastrozole [84]. Additionally, one short-term study did not show adverse effects of aromatase inhibition in older men on cardiovascular markers. However, it is not clear that this conclusion also holds for boys: vertebral deformities were observed in boys treated for delayed onset of puberty [85]. Furthermore, hyperandrogenism induced by treatment with aromatase inhibitors may result in decreased HDL-cholesterol and increased hemoglobin levels [86], indicating the need for follow-up during treatment. The same group of investigators concluded that there were no effects of letrozole on cognitive performance could be detected in a group of prepubertal boys [87]. In a group of elderly men who obtained exogenous testosterone enenthate, the addition of anastrozole to the injected androgen prevented the androgen induced improvement of verbal memory, but did not affect special memory [88].

## Conclusion

Over the years compelling evidence has accumulated that in men estradiol has an important role in gaining and maintaining bone mass, closing of the epiphyses and feedback on gonadotrophin release. Aromatase inhibitors, mostly combined with agonists of gonadotrophin-releasing hormone proved effective for the prevention of premature epiphysial closure in boys with pubertas praecox of various etiologies. There is also evidence that aromatase inhibitors can be used in boys with idiopathic short stature and boys with constitutional delay of puberty to increase adult height. Aromatase inhibitors are not effective for the treatment of gynecomastia in pubertal boys and have limited efficacy for the prevention of gynecomastia in bicalutamide-treated men with prostate cancer. Although aromatase inhibitors increase FSH levels, there is no consistent evidence for a beneficial effect on spermatogenesis. In older men with so-called late-onset hypogonadism, aromatase inhibitors may emerge as an attractive alternative for traditional testosterone supplementation to improve testosterone levels. The long-term benefits of higher testosterone levels in older men remain controversial, however. Moreover, it is questionable whether aromatase inhibitors are able to stimulate testosterone production sufficiently in men with truly low testosterone levels for whom testosterone treatment is currently recommended. Although most of the recent studies with aromatase inhibitors in boys and adult men do not show major detrimental effects on bone long-term skeletal safety remains an issue of concern.

## Competing interests

The authors declare that they have no competing interests.

## Authors' contributions

WDR wrote the original version of this manuscript. FHDJ extended the text with more recent observations and conclusions. Both authors read and approved the final manuscript.
